# Profiling of Somatic Mutations in Phaeochromocytoma and Paraganglioma by Targeted Next Generation Sequencing Analysis

**DOI:** 10.1155/2015/138573

**Published:** 2015-03-25

**Authors:** Andrea Luchetti, Diana Walsh, Fay Rodger, Graeme Clark, Tom Martin, Richard Irving, Mario Sanna, Masahiro Yao, Mercedes Robledo, Hartmut P. H. Neumann, Emma R. Woodward, Farida Latif, Stephen Abbs, Howard Martin, Eamonn R. Maher

**Affiliations:** ^1^Department of Medical Genetics, University of Cambridge, Cambridge Biomedical Campus, Cambridge CB2 0QQ, UK; ^2^Cambridge NIHR Biomedical Research Centre, Addenbrooke's Hospital, Cambridge CB2 0QQ, UK; ^3^Centre for Rare Diseases and Personalised Medicine, University of Birmingham, Birmingham B15 2TT, UK; ^4^Queen Elizabeth Hospital, Queen Elizabeth Medical Centre, Birmingham B15 2TH, UK; ^5^Department of Otology & Skull Base Surgery, Gruppo Otologico, Via Antonio Emmanueli 42, 29121 Piacenza, Italy; ^6^Department of Medical, Oral and Biotechnological Sciences, G. d'Annunzio University, Via dei Vestini 1, 66100 Chieti, Italy; ^7^Department of Urology, Yokohama City University School of Medicine, 3-9 Fukuura, Kanazawa Ward, Yokohama, Kanagawa 236-0004, Japan; ^8^Hereditary Endocrine Cancer Group, Spanish National Cancer Research Centre (CNIO), Madrid, Spain; ^9^ISCIII Center for Biomedical Research on Rare Diseases (CIBERER), Madrid, Spain; ^10^Section of Preventive Medicine, Department of Nephrology, Albert Ludwigs University of Freiburg, Hugstetter Strasse 55, 79106 Freiburg, Germany; ^11^Department of Clinical Genetics, Birmingham Women's Hospital, Birmingham B15 2TG, UK

## Abstract

At least 12 genes (*FH, HIF2A, MAX, NF1, RET, SDHA, SDHB, SDHC, SDHD, SDHAF2, TMEM127,* and * VHL*) have been implicated in inherited predisposition to phaeochromocytoma (PCC), paraganglioma (PGL), or head and neck paraganglioma (HNPGL) and a germline mutation may be detected in more than 30% of cases. Knowledge of somatic mutations contributing to PCC/PGL/HNPGL pathogenesis has received less attention though mutations in *HRAS, HIF2A, NF1, RET,* and *VHL* have been reported. To further elucidate the role of somatic mutation in PCC/PGL/HNPGL tumourigenesis, we employed a next generation sequencing strategy to analyse “mutation hotspots” in 50 human cancer genes. Mutations were identified for *HRAS* (c.37G>C; p.G13R and c.182A>G; p.Q61R) in 7.1% (6/85); for *BRAF* (c.1799T>A; p.V600E) in 1.2% (1/85) of tumours; and for *TP53* (c.1010G>A; p.R337H) in 2.35% (2/85) of cases. Twenty-one tumours harboured mutations in inherited PCC/PGL/HNPGL genes and no *HRAS, BRAF*, or *TP53* mutations occurred in this group. Combining our data with previous reports of *HRAS* mutations in PCC/PGL we find that the mean frequency of *HRAS/BRAF* mutations in sporadic PCC/PGL is 8.9% (24/269) and in PCC/PGL with an inherited gene mutation 0% (0/148) suggesting that *HRAS/BRAF* mutations and inherited PCC/PGL genes mutations might be mutually exclusive. We report the first evidence for *BRAF* mutations in the pathogenesis of PCC/PGL/HNPGL.

## 1. Introduction

Phaeochromocytoma (PCC) and paraganglioma (PGL) are neuroendocrine tumours deriving from chromaffin cells of the medulla of the adrenal glands or from extra-adrenal chromaffin tissue like ganglia of the sympathetic nervous system, respectively. Approximately 10% of tumours are malignant but the most common presentation results from the cardiovascular effects of catecholamine hypersecretion that causes hypertension, tachycardia, excessive sweating, and/or anxiety. The majority of PCC/PGL occur sporadically but although only about 10% of cases have a family history, more than a third of cases harbour a germline mutation in one of the 12 inherited PCC/PGL genes (*FH, HIF2A, MAX, NF1, RET, SDHA, SDHB, SDHC, SDHD, SDHAF2, TMEM127, *and* VHL*) that have been shown to be mutated in multiple families and some of these genes are also somatically mutated in sporadic PCC/PGL [1–8]. Germline mutations in most of these genes may also cause head and neck paraganglioma (HNPGL) which are derived from parasympathetic nervous system ganglia and are nonsecretory [9–12]. It appears that disruption of multiple cellular signalling pathways is implicated in PCC/PGL/HNPGL tumourigenesis. Thus known inherited predisposition gene products belong to multiple functional classes including kinase receptor and signalling regulators (*RET* and* NF1*), transcription factors (*MAX*), energy metabolism components (*FH*,* SDH*-subunits* A*,* B*,* C*, and* D*, and cofactor* SDHAF2*), constituents of the cellular response to hypoxia (*VHL* and* HIF2A/EPAS1*), and endosomal signalling (*TMEM127*) [1–8, 13–15]. Nevertheless, gene expression studies have suggested that most PCCs and PGLs can be classified into two distinct groups (cluster 1 and cluster 2) by transcription profiling: cluster 1 includes tumours that harbour mutations in genes linked to the hypoxic gene response (*VHL, HIF2A, SDHA, SDHB, SDHC, *and* SDHD*) and cluster 2 contains tumours harbouring mutations in genes that are involved in the kinase signalling characterized by the activation of the PIK3/AKT/mTOR and RAS/RAF/ERK pathways (*RET, NF1, TMEM127, MAX*, and* HRAS*) [14–18].

The last 10 years have seen considerable progress in identifying the genetic basis of inherited PCC/PGL/HNPGL, but relatively less progress has been made with respect to understanding the somatic mutations that underlie tumour initiation and progression in these tumour types. Somatic mutations in inherited PCC/PGL genes can be detected in ~25–30% of the sporadic tumours (mainly involving* RET, VHL, NF1, MAX*, and* HIF2A* genes). Recently, an exome resequencing study led to the identification of somatic* HRAS* activating mutations in sporadic PCC/PGLs [19]. Subsequently, this finding was confirmed and the overall frequency of somatic* HRAS* mutations has been estimated at about 7% [20]. However, to date* HRAS* analysis in PCC/PGL has mostly been performed by Sanger sequencing and the frequency of* HRAS* mutations might have been underestimated because of the lower sensitivity of Sanger sequencing analysis, compared to next generation sequencing approaches, to detect mosaic mutations [21]. Furthermore, in contrast to many other tumour types, there is little information available on many of the genes most commonly mutated in human neoplasia. We therefore investigated, in tumour samples, the frequency of mutations in critical regions of 50 human cancer genes by next generation sequencing in a large series (*n* = 85) of inherited and sporadic PCC/PGL/HNPGL.

## 2. Materials and Methods

### 2.1. Subjects

Tumour material from 85 patients with PCC (*n* = 60), PGL (*n* = 5), or HNPGL (*n* = 20) was collected for analysis. 21 patients were known to harbour a germline inherited gene mutation (*VHL *= 10,* RET *= 3,* NF1 *= 1,* SDHB *= 5,* SDHC *= 1, and* SDHD *= 1) and 64 cases were sporadic. All patients gave informed consent and the study was approved by the South Birmingham Ethics Committee.

### 2.2. Next Generation Sequencing (NGS)

#### 2.2.1. Technical Assessment

To investigate the sensitivity of somatic mutation detection, a dilution series of two well characterized DNA samples was created. A sample bearing* HRAS* (c.81T>C) and one a* PIK3CA* (c.1173A>G) were titrated together to create serial dilutions containing allelic frequencies ranging from 50% to 0.1%. Each dilution was amplified using the Ion AmpliSeq Cancer Hotspot Panel v2 (Life Technologies, UK). Amplicons underwent library preparation according to protocol. Before emulsion PCR each point of the titration curve was identified by using a different Ion Xpress Barcode. Subsequently library was run on 318 chip v2 (Life Technologies, UK) on the Ion PGM (Life Technologies, UK). The output reads from the chip were processed using the Torrent browser suite software (v.4.0.2).

#### 2.2.2. Sample Sequencing

DNA was isolated from both tumour material and peripheral blood using standard methodology. Genomic DNA (gDNA) samples were quality-checked on DNA NanoDrop 1000 considering acceptable absorbance ratio greater than 1.7 for both 260/280 and 230/260 nm. Each sample was then quantified with the Qubit2.0 fluorometer (Life Technologies, UK) by using the Quant-IT dsDNA BR Assay (Life Technologies, UK). For the AmpliSeq Library, 10 ng of gDNA was used for library generation. Libraries were indexed using the Ion Xpress Barcode Adapter Kit and quantified using the Quant-IT dsDNA HS Assay (Life Technologies, UK) on Qubit 2.0. Appropriate dilutions were performed based on amplicon concentration at the 80–125 bp range. Twenty pM of individual indexed amplicon libraries were pooled for emulsion PCR and 16 samples were sequenced on the Ion Torrent PGM platform using the 318 v2 chip (Life Technologies, UK). Mean coverage for each sample was over 1000x. Sequence reads were mapped against the human reference genome (hg19) with the Torrent Mapping Alignment Program (TMAP) using the default software settings. Output was restricted to the targeted regions as defined by the sequence capture design BED file, and SNPs and INDELs were characterized as being significantly different from the reference sequence if the variant to reference base frequency was greater than 5%. All identified variants within a particular sample were saved as variant call format file (VCF version 4.1). VCFs were examined with the online tool “Ingenuity Variant Analysis” (Qiagen) for variant annotation and prediction of variant effects on genes. In addition, BAM files were inspected manually in order to remove likely artefactual variants (i.e., close to homopolymers) and to detect any mutations in known driver genes, especially insertions and deletions that were not called by the Ion Torrent software.

### 2.3. Sanger Sequencing

To confirm NGS results, fragments of hotspot codons in* HRAS* (exons 2 and 3),* BRAF* (exon 15), and* TP53 *(exon 10) were amplified, in both tumour and constitutional (blood) DNA, by PCR and sequenced with automated Sanger sequencing. Primers sequences and PCR conditions are available on request.

### 2.4. Statistical Analysis

Patients with mutated* HRAS* and* BRAF* were compared to patients with negative genetic screening.

*P* values <0.05 were considered statistically significant.

## 3. Results

### 3.1. Technical Assessment of Next Generation Sequencing Assay

The analytical sensitivity of the AmpliSeq Hotspot panel was determined using serial dilutions of tumour DNA carrying* PIK3CA* and* HRAS *mutations. This demonstrated that it was possible to reliably detect mutations at 1% allele frequency (data not shown). Sequence coverage was assessed considering the number and distribution of reads that were present in the target DNA regions. Each sample had approximately 317000 mapped reads with a mean read length of 107 bp that generates approximately 23 Mb of sequence with depth of coverage of 1400 reads.

### 3.2. Detection of Mutations in Inherited PCC/PGL/HNPGL Genes

21 patients were known to harbour a germline mutation in an inherited PCC/PGL gene (*VHL *= 10,* RET *= 3,* NF1 *= 1,* SDHB *= 5,* SDHC *= 1, and* SDHD *= 1). 13 of these mutations (*RET* (*n* = 3) and* VHL* (*n* = 10)) were detected by NGS in 13 tumours (see Table 1) and 8 patients had a clinical or previous molecular diagnosis of a germline inherited PCC/PGL gene mutation (*NF1* (*n* = 1),* SDHB* (*n* = 5),* SDHC* (*n* = 1), and* SDHD* (*n* = 1)) that was not covered by the NGS assay. No mutations in inherited PCC/PGL genes were detected in the 64 sporadic tumours.

### 3.3. Detection of Activating Mutations in Protooncogenes and Tumour Suppressor Genes

Six tumours (PCC = 6/60, PGL = 0/5, and HNPGL = 0/20) harboured an activating missense mutation in the* HRAS* hotspot region of codons 13 and 61 (c.37G>C; p.G13R = 1 and c.182A>G; p.Q61R = 5), giving an overall frequency of 7.1% (6/85, 95% CI = 2.63%–14.73%). In each case the somatic status of the mutations was confirmed when the detected mutation was absent in matched constitutional DNA (blood) (Table 2). In one PCC tumour (1/85) an activating* BRAF *mutation was identified (c.1799T>A; p.V600E, 1.2%, 95% CI = 0%–6.38%) (Table 2). In two tumours a missense mutation (c.1010G>A; p.R337H, 2/85, 2.4%, 95% CI = 0.29%–8.2%) occurring in the tetramerisation domain of TP53 protein was identified (Table 2).

### 3.4. Exclusion of Mutations in Protooncogene Hotspots

No mutations were detected at hotspot mutation regions in 11 oncogenes frequently mutated in human cancer (*AKT, MET, PIK3CA, KRAS, NRAS, IDH1, IDH2, NOTCH, SMO, ABL*, and* EGFR*).

### 3.5. Relationship between Clinical Status and* HRAS* Mutations

We investigated the relationships between* HRAS* mutation and tumour location and presence or absence of an inherited PCC/PGL/HNPGL gene mutation. The frequency of* HRAS* mutations in PCC, PGL, and HNPGL was 6/60 (10%, 95% CI = 3.76%–20.51%), 0/5 (0%, 95% CI = 0%–52.18%), and 0/20 (0%, 95% CI = 0%–16.84%), respectively. Among 21 tumours from patients with known inherited disease and/or detectable mutation in inherited PCC/PGL/HNPGL genes there were no* HRAS* mutations (0%, 95% CI = 0%–16.11%) whereas a mutation was present in 6/64 (9.4%, 95% CI = 3.52%–19.30%) of patients without a clinical diagnosis of inherited PCC/PGL/HNPGL or a detectable mutation in an inherited PCC/PGL/HNPGL gene (*P* = 0.33).

To further investigate possible relationships between these attributes we combined our data for* HRAS *mutation status with 18 mutations in 353 PCC/PGL/HNPGL from two previously published studies (Tables 3 and 4) [19, 20]. Meta-analysis results showed that the overall prevalence of* HRAS *mutations in the cohort of PCC/PGL is 5.48% (24/438, 95% CI = 3.54%–8.04%) increasing to 8.9% (24/269, 95% CI = 5.80%–12.98%) considering cases without an inherited PCC/PGL gene mutation and to 9.87% (23/233, 95% CI = 6.36%–14.44%) considering only PCC samples without an inherited PCC/PGL gene. In PCC/PGL with an inherited gene mutation the* HRAS* mutation frequency was 0% (0/148, 95% CI = 0%–2.46%) (PCC/PGL unknown mutation versus PCC/PGL known mutation, *P* = 0.0001).

## 4. Discussion

**Figure 1:**
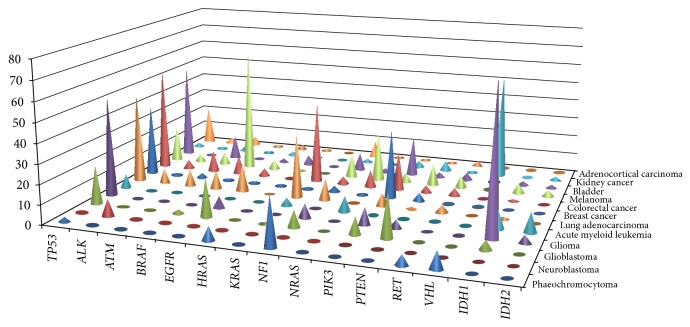
Comparison of somatic variant frequencies in multiple cancer genes in different cancer types.

A wide repertoire of genetic and epigenetic events can be implicated in human neoplasia. Previous studies have demonstrated that tumour suppressor gene (TSG) inactivation and oncogene activation in PCC/PGL may result from somatic copy number abnormalities (SCNA), intragenic mutations, and epigenetic silencing of transcription by promoter methylation [22]. Common copy number changes in PCC include loss of chromosomes 1p, 3q, 3p, 11p, 11q, 6q, 17p, and 22 [23–27] and gain of chromosomes 9q, 17q, 19p13.3, and 20q [28, 29]. Epigenetic inactivation of candidate TSGs has been reported relatively frequently in PCC/PGL. Thus promoter methylation of candidate TSGs including* RASSF1A, FLIP, TSP1, DCR1, DCR2, DR4, DR5, CASP8*, and* HIC1* was reported at a frequency of >20% of tumours analysed [30–32]. Until recently, investigations of patterns of somatic mutations in PCC/PGL/HNPGL had concentrated on analysing genes known to be associated with inherited PCC/PGL/HNPGL. Thus* NF1, VHL, RET*, and* MAX* germline and somatic mutations have been reported in 3–25%, 13–9%, 5–5%, and 1–3% of tumours, respectively [33]. Recently activating mutations in* HIF2A* and* HRAS* were reported in a subset of these tumours [6, 7, 19, 20]. Whilst* HIF2A* mutations may be found in multiple tumours from patients without a detectable germline mutation (suggesting low level constitutional mosaicism) or occasionally as a germline mutation [5], to date* HRAS* mutations have only been detected as somatic changes (germline* HRAS *mutations are associated with Costello syndrome but PCC/PGL/HNPGL are apparently not a feature of this disorder) [34]. The frequency of* HRAS* mutations in our cohort was similar to that in other recent studies.* HRAS* mutation hotspots at codons 13 and 61 affect the RAS GTP hydrolysis domain, leading to a constitutive activated state with resistance to upstream inhibitory proteins, such as neurofibromin (*NF1* gene product). This overactive RAS signalling leads to increased activity of downstream effectors, most notably the RAS/RAF/ERK and PI3K/AKT/mTOR signalling pathways linked to increased cell proliferation and tumour formation [14–18]. The identification of* HRAS* mutations as a new pathogenetic driver in sporadic PCC opens up the possibility of new therapeutic approaches—though in most cases surgical removal seems likely to be the treatment of choice.* BRAF* mutations are found in multiple cancer types (Figure 1), notably those that are also associated with mutations in isoforms of RAS (i.e., malignant melanoma, colorectal cancer). Our results demonstrate for the first time a somatic* BRAF *mutation in PCC/PGL/HNPGL samples. The mutation detected is the most common* BRAF* mutation found in human neoplasia and results from a T to A transversion at nucleotide 1799. c.1799T>A (p.V600E) mutant BRAF proteins are characterized by an increased kinase activity and have been demonstrated to induce cellular transformation in* in vitro* studies [35].

Since BRAF is involved in ERK kinase activation in the RAS/RAF/ERK signalling pathway, it will be interesting to further compare the biological behaviour of* HRAS *and* BRAF *mutated tumours in order to determine whether they have similar clinical characteristics and to determine if they will fall into the “cluster 2” group of gene expression patterns. PCC/PGLs with* BRAF* V600E mutations may be predicted to respond to BRAF and MEK inhibitors, such as vemurafenib and trametinib/selumetinib, better than wild type* BRAF *tumours [36–39].

The presence of* BRAF* and* HRAS* mutations in PCC/PGL suggests that activation of the RAS/RAF/ERK signalling pathway can be triggered by mutations at various levels in the pathway. An implementation of the panel used, including more genes of this pathway, could be useful to identify low frequency somatic variants in PCC/PGL. Loss of heterozygosity at 17p13 is frequent in many tumour types including breast [40], colon [41], and hepatocellular cancers [42] and phaeochromocytoma [22], with an occurrence ranging from 30% to 60%. The tumour suppressor gene* TP53 *maps in this region and has been demonstrated to be implicated in the tumourigenesis process in different types of cancers. Loss of TP53 function could arise from epigenetic alterations allelic losses and mutational events. Due to its involvement in carcinogenesis,* TP53 *has been intensively studied in PCC/PGL but, despite frequent allele loss,* TP53 *mutations have been reported rarely [23, 43–45]. However, in our series, NGS results followed by direct DNA sequencing demonstrated the presence in two sporadic PCC samples of a somatic* TP53* gene mutation (c.1010G>A; p.R337H) (Table 2). The R337H substitution has previously been extensively characterised as a founder germline mutation and the altered protein demonstrated to act as a conditional mutant that loses its function only when a small increase in intracellular pH occurs in cells. Initially, the c.1010G>A (R337H) mutation was thought to predispose only to adrenal cortical carcinoma (ACC) (for which the penetrance of the allele has been estimated to be ~10% and the increased risk was estimated at a 20,000-fold increase [46, 47]); however, several studies have highlighted it in association with Li Fraumeni-like syndrome [48], breast cancer [49, 50], choroid plexus carcinoma, and osteosarcoma [51, 52]. To our knowledge, this is the first report on this mutation in sporadic phaeochromocytoma. By using an approach utilising targeted deep sequencing we were able to confidently detect the presence or absence of a large repertoire of hotspot mutations, paying particular attention to a set of them. The absence of mutations in* ALK *and* NRAS *was of particular interest (Figure 1) because mutations in these genes have been described in neuroblastoma (~9.2%, 0.83% of cases, resp.; Figure 1) and some epigenetically inactivated candidate TSGs (i.e.,* RASSF1A, FLIP, CASP8*, and* HIC1*) are common to both PCC/PGL and neuroblastoma.* IDH1* and* IDH2* mutations have been described in gliomas, leukaemia, and other malignancies (Figure 1) and may cause methylation abnormalities by a similar mechanism to that associated with* SDH* gene subunit mutations [53]. Therefore despite* IDH1/IDH2* hotspot regions potentially representing candidate genes for somatic inactivation in PCC/PGL/HNPGL, no mutations were identified (Figure 1) [54]. Our findings are consistent with the hypothesis that mutations in* HRAS *and inherited PCC/PGL genes are mutually exclusive driver mutations (though comprehensive analysis of all inherited phaeochromocytoma/PGL genes has not been undertaken in all patients). If this hypothesis is correct, it can be suggested that* HRAS* profiling of PCC/PGL/HNPGL tumour material could aid the management of patients by enabling enhanced stratification of their risk of inherited disease. Furthermore, we suggest that molecular profiling should be expanded to include* BRAF* analysis. Currently histopathology cannot reliably predict the likelihood of malignancy in PCC/PGL/HNPGL whereas the presence of a germline* SDHB *mutation is associated with an increased risk of malignancy [55–57]. The identification, careful characterisation, and follow-up of cohorts of patients with* HRAS/BRAF* mutation positive tumours could enable the natural history of such tumours (e.g., absence of malignant or recurrent disease) and so facilitate personalised management of patients with these tumours.

## Figures and Tables

**Table 1:** Mutations detected in inherited PCC/PGL/HNPGL genes.

Tumour ID	Type of tumour	Gene	Mutation type
P1	PCC	*RET *	c.1900T>C
P2	PCC	*RET *	c.1901G>A
P3	PCC	*RET *	c.2753T>C
P4	PCC	*VHL *	c.241C>T
P5	PCC	*VHL *	c.292T>C
P6	PCC	*VHL *	c.292T>C
P7	PCC	*VHL *	c.292T>C
P8	PCC	*VHL *	c.292T>C
P9	PCC	*VHL *	c.292T>C
P10	PCC	*VHL *	c.292T>C
P11	PCC	*VHL *	c.292T>C
P12	PCC	*VHL *	c.292T>C
P13	PCC	*VHL *	c.374A>C
P14	PCC	*SDHB *	c.470delT

**Table 2:** Oncogene mutations identified in next generation sequencing analysis of 85 PCC/PGL/HNPGL.

Tumour ID	Clinical diagnosis	Type of tumour	Gene	Codon change	Aminoacid change	Allele frequency
P19	Phaeochromocytoma	PCC	*HRAS *	c.37G>C	G13R	72%
P20	Phaeochromocytoma	PCC	*HRAS *	c.182A>G	Q61R	36%
P21	Phaeochromocytoma	PCC	*HRAS *	c.182A>G	Q61R	27%
P22	Phaeochromocytoma	PCC	*HRAS *	c.182A>G	Q61R	40%
P23	Phaeochromocytoma	PCC	*HRAS *	c.182A>G	Q61R	50%
P24	Phaeochromocytoma	PCC	*HRAS *	c.182A>G	Q61R	26%
**P25**	**Phaeochromocytoma**	**PCC**	***BRAF***	**c.1799T>A**	**V600E**	**10%**
**P26**	**Phaeochromocytoma**	**PCC**	***TP53***	**c.1010G>A**	**R337H**	**4%**
**P27**	**Phaeochromocytoma**	**PCC**	***TP53***	**c.1010G>A**	**R337H**	**21%**

**Table 3:** Meta-analysis of *HRAS* mutations in phaeochromocytoma, paraganglioma, and HNPGL.

Study	Frequency of *HRAS* mutations in phaeochromocytoma					Frequency of *HRAS* mutations in paraganglioma					Frequency of *HRAS* mutations in HNPGL				
	All	Positive samples	Sporadic	Positive samples	Inherited	All	Positive samples	Sporadic	Positive samples	Inherited	All	Positive samples	Sporadic	Positive samples	Inherited
Current study	60	**6**	44	0	16	5	**0**	5	0	0	20	**0**	15	0	5
Crona et al. [19]	72	**3**	50	0	22	9	**1**	6	0	3	1	**0**	1	0	0
Oudijk et al. [20]	216	**14**	140	0	76	55	**0**	24	0	31	0	**0**	0	0	0

Total	348	***23***	234	0	114	69	***1***	35	0	34	21	***0***	16	0	5

**Table 4:** *HRAS* mutations frequencies in phaeochromocytoma and paraganglioma.

Study	Overall frequency of *HRAS* mutations			Frequency of *HRAS* in PCC/PGL with unknown mutations			Frequency of *HRAS* in PCC with unknown mutations		
		All	Positive samples		All	Positive samples		All	Positive samples
Current study	7,06%	**85**	6	12,24%	**49**	6	13,95%	**43**	6
Crona et al. [19]	4,88%	**82**	4	7,14%	**56**	4	6,00%	**50**	3
Oudijk et al. [20]	5,17%	**271**	14	8,54%	**164**	14	10,00%	**140**	14

Total	5,48%	***438***	24	8,92%	***269***	24	9,87%	***233***	23
